# Common pitfalls during model specification in psychophysiological interaction analysis

**DOI:** 10.1162/IMAG.a.989

**Published:** 2025-11-10

**Authors:** Vicky He, Bahman Tahayori, David N. Vaughan, Heath R. Pardoe, Graeme D. Jackson, Chris Tailby, David F. Abbott

**Affiliations:** The Florey Institute of Neuroscience and Mental Health, Heidelberg, Victoria, Australia; Florey Department of Neuroscience and Mental Health, The University of Melbourne, Parkville, Victoria, Australia; Department of Neurology, Austin Health, Heidelberg, Victoria, Australia; Department of Clinical Neuropsychology, Austin Health, Heidelberg, Victoria, Australia; Department of Medicine - Austin Health, The University of Melbourne, Heidelberg, Victoria, Australia

**Keywords:** fMRI, psychophysiological interaction, functional connectivity, prewhitening, mean-centering

## Abstract

Psychophysiological interaction (PPI) analysis is a widely used regression method in functional neuroimaging for capturing task-dependent changes in connectivity from a seed region. The present work identifies, and provides corrections for, common methodological pitfalls in PPI analysis that compromise model validity. Firstly, if the seed time series is extracted with prewhitening, the temporal structure of the signal is altered and subsequent deconvolution of prewhitened data becomes suboptimal. Furthermore, prewhitening again during model fitting results in double prewhitening of the seed regressor. Secondly, a failure to mean-center the task regressor when calculating the interaction term can also lead to model misspecification and potentially spurious inferences. By using simulations and empirical language fMRI data from the Australian Epilepsy Project, we demonstrate the adverse effects of these issues, and how they are resolved when corrected. A systematic review of current practices revealed widespread model misspecification, and underreporting of methods, in published PPI studies. We provide clearer reporting guidelines, and advocate for appropriate methods for handling of prewhitening and mean-centering to ensure the validity of PPI analyses.

## 1 Introduction

Psychophysiological interaction (PPI) analysis is a multiple regression neuroimaging analysis method that estimates task-dependent changes in the functional connectivity from a seed region to other brain areas (Friston et al., 1997). Despite its widespread use, the correct specification of the PPI model has been a topic of considerable debate (e.g., Di et al., 2017; Gitelman et al., 2003; Masharipov et al., 2024; O’Reilly et al., 2012). In this paper, we focus on PPI analyses of functional magnetic resonance imaging (fMRI) data. We examine the implications of two model specification steps on model performance: (1) prewhitening and (2) mean-centering. We assess how these steps influence group-level analyses, including group mean comparisons and behavioral regressions.

In a system modeled by PPI, the relevant interactions of interest occur at the neuronal level rather than the level of the haemodynamic responses. To capture the neuronal response, and model its interaction with the psychological variable, one can deconvolve the haemodynamic response function (HRF) from the seed time course (Gitelman, 2003). This has been shown to increase sensitivity in PPI studies, in both block and event-related designs (Di & Biswal, 2017; Masharipov et al., 2024). It has also been shown in a simulation study that only with deconvolution can PPI models incorporating a three-level task (generalized PPI) estimate the direction of information flow (Masharipov et al., 2024).

**Fig. 1.**
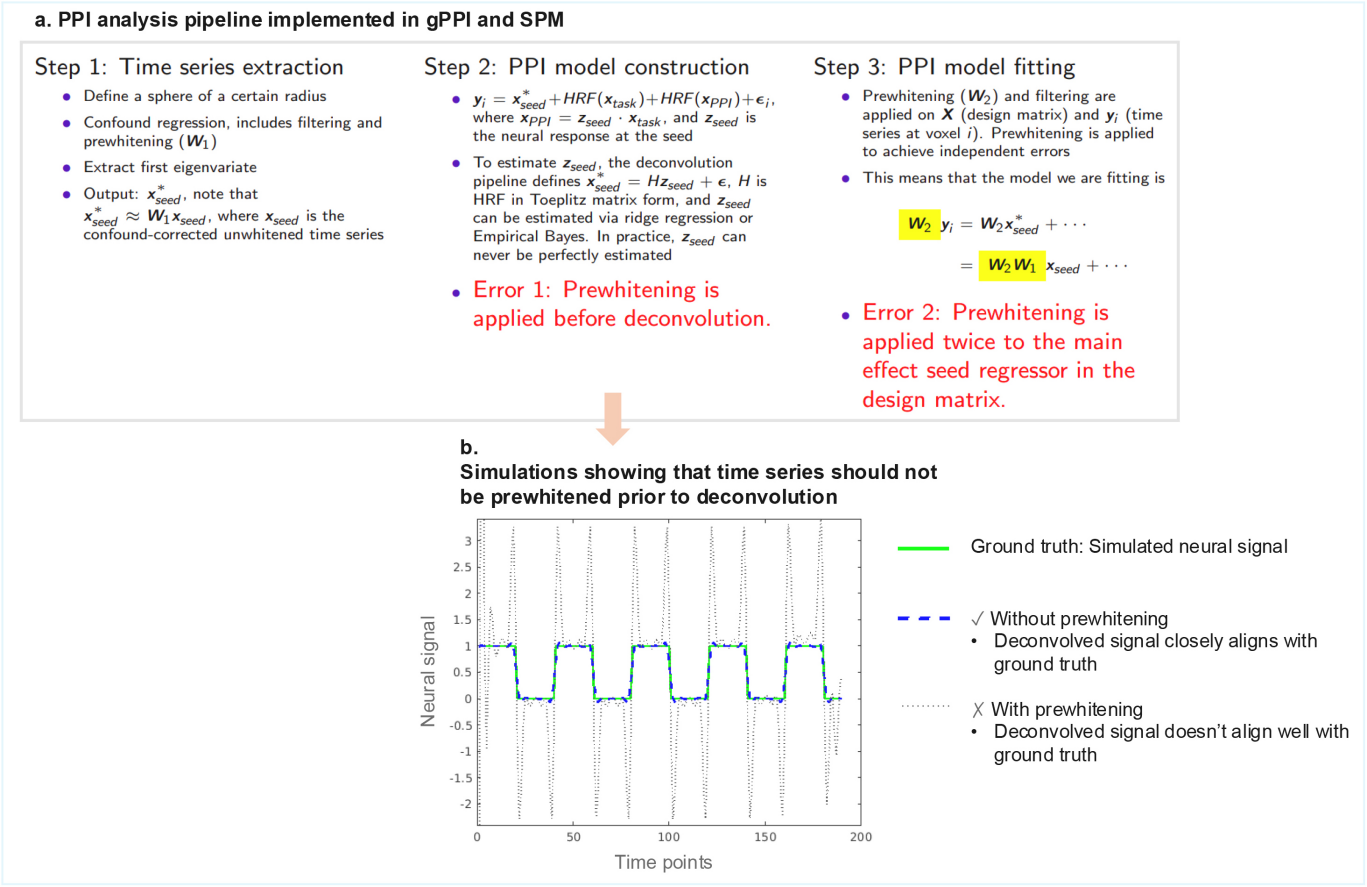
(a) The PPI processing pipeline in SPM and gPPI has two issues associated with prewhitening during time series extraction. The first arises during the construction of the interaction (PPI regressor), as deconvolution is applied to a prewhitened time series. The second issue occurs during model fitting, where prewhitening is applied again to the design matrix, resulting in the main effect seed time series being prewhitened twice. (b) A simulated example showing that time series should not be prewhitened prior to deconvolution, as prewhitening alters the structure of the time series. The deconvolved signal with prewhitening (grey dotted line) has been scaled and vertically shifted to better match the ground truth.

The above approach can be carried out in the SPM software (Friston et al., 2007), the generalized PPI toolbox (gPPI; McLaren et al., 2012), and the task-modulated functional connectivity toolbox (TMFC; Masharipov et al., 2024). One typically first extracts a time series of a seed region using SPM’s time series extraction function. This function fits a general linear model (GLM) to all voxels within the region of interest (ROI), so as to remove the effects of confound regressors such as realignment parameters. The first eigenvariate of the residuals are then extracted. In this process, the extracted time series is prewhitened (see Fig. 1a) to ensure that when regressing out confounds, the errors are independent. While prewhitening may be sensible to ensure valid inference at the confound correction stage, we argue that it adversely affects two downstream processes. Firstly, the subsequent deconvolution step will be applied to a prewhitened time series, potentially biasing the estimation of the underlying neuronal response. Secondly, the seed regressor that enters the PPI model design matrix is already prewhitened. Thus, when prewhitening is applied during the actual fitting of the PPI model, the seed regressor is effectively prewhitened a second time, causing it to depart from the seed time course it intended to capture (Fig. 1a). We refer to this as the double prewhitening issue. As a solution, we propose multiplying the extracted seed time series by the inverse of the whitening matrix (**W**^-1^) before downstream processing. We refer to this process as whitening inversion.

Another issue confounding PPI analyses concerns (lack of) mean-centering of the task regressor during creation of the interaction term, as well explained by Di et al. (2017). Due to the necessarily imperfect deconvolution, without mean-centering the interaction term carries unwanted correlation with the main effect of the seed. The model might incorrectly attribute this correlation to the interaction term, resulting in a spuriously inferred interaction effect (Di et al., 2017). To resolve this issue, one has to mean-center the task regressor when calculating the interaction term (Di et al., 2017; Masharipov et al., 2024). For simplicity, we refer to this as ‘mean-centering’ in this paper.

However, a widely used software package for PPI analysis, the gPPI toolbox, with its most recent update in 2014 (version 13.1), has not incorporated the mean-centering approach advocated by Di et al. (2017). Previously, we modified the gPPI toolbox to apply PPI analysis with mean-centering to estimate task-dependent connectivity in the reading network (He et al., 2025). Here, we use this same dataset to extend the work of Di et al. (2017) to investigate how prewhitening and non-mean-centering affect group comparisons and behavioral correlations in PPI analyses. In addition, we systematically reviewed PPI literature published since 2017 to determine whether mean-centering has become a new standard in the field. Finally, in the discussion, we provide recommendations for the implementation, and reporting of PPI studies.

## 2 Methods

### 2.1 Simulations of prewhitening prior to deconvolution

To illustrate the effect of prewhitening before deconvolution, we first performed a simple simulation. We generated a time series resembling a block design (green solid line in Fig. 1b) and convolved it with SPM’s canonical HRF to approximate a BOLD response. We then applied two approaches: (1) direct deconvolution of the time series and (2) prewhitening and then deconvolution. For simplicity, we estimated the whitening matrix by fitting an AR(1) model using MATLAB’s *arima* command. In the simulations, deconvolution was performed using ridge regression with a shrinkage parameter of 0.002, as this value approximates SPM’s built-in Empirical Bayes procedure (Masharipov et al., 2024).

### 2.2 Participants

Participant characteristics have been described in He et al. (2025). Briefly, the study included 94 adult participants with seizure disorders (median age 33, interquartile range = 19; 47 males) and 107 healthy controls (median age 43, interquartile range = 22; 38 males) from the Australian Epilepsy Project (AEP). The study was approved by the Austin Health Human Research Ethics Committee (HREC/60011/Austin-2019 and HREC/68372/Austin-2022).

### 2.3 MRI data acquisition, preprocessing, and fMRI paradigm

We collected T1-weighted as well as Multi-Band Multi-Echo (MBME) fMRI images for all participants in a 3T Siemens PrismaFit MRI scanner. Details of scanning parameters and preprocessing are discussed in our previously published paper (He et al., 2025). All participants completed a block design language fMRI task. In the task-active phase, participants decide whether visually presented pairs of pseudowords rhyme or not. In the baseline phase, participants decide whether pairs of patterns of forward and backward slashes are identical. Each stimulus pair was displayed for 4.5 s, and each block consisted of four stimulus pairs. The experiment lasted 180 s (TR = 0.9 s; 200 TRs in total) and included 20 rhyming and 20 pattern matching pairs. The fMRI paradigm does not have a separate resting baseline.

### 2.4 Task activation analysis

We first fitted a GLM to contrast the pseudoword rhyming blocks (coded as 1) against the pattern matching baseline (coded as 0). Confound regressors included 24 head motion parameters (Friston et al., 1996), the first eigenvariates of white matter and cerebrospinal fluid signals, and a constant term. The model also incorporated a 128-s high-pass filter and prewhitening with a FAST model, recommended for TR shorter than 1 s (Corbin et al., 2018; Olszowy et al., 2019). One-sample *t*-tests were conducted on individual first-level task activation estimates (n = 201). A two-tailed Family-Wise-Error cluster corrected (FWEc) threshold of p < 0.05 was applied, with an initial cluster-forming threshold of p < 0.001 uncorrected. Task activation analysis was performed using the iBT software version 3.9 (Abbott et al., 2024) with SPM12 revision 7771 (Friston et al., 2007).

### 2.5 Task-modulated connectivity analysis

**Fig. 2.**
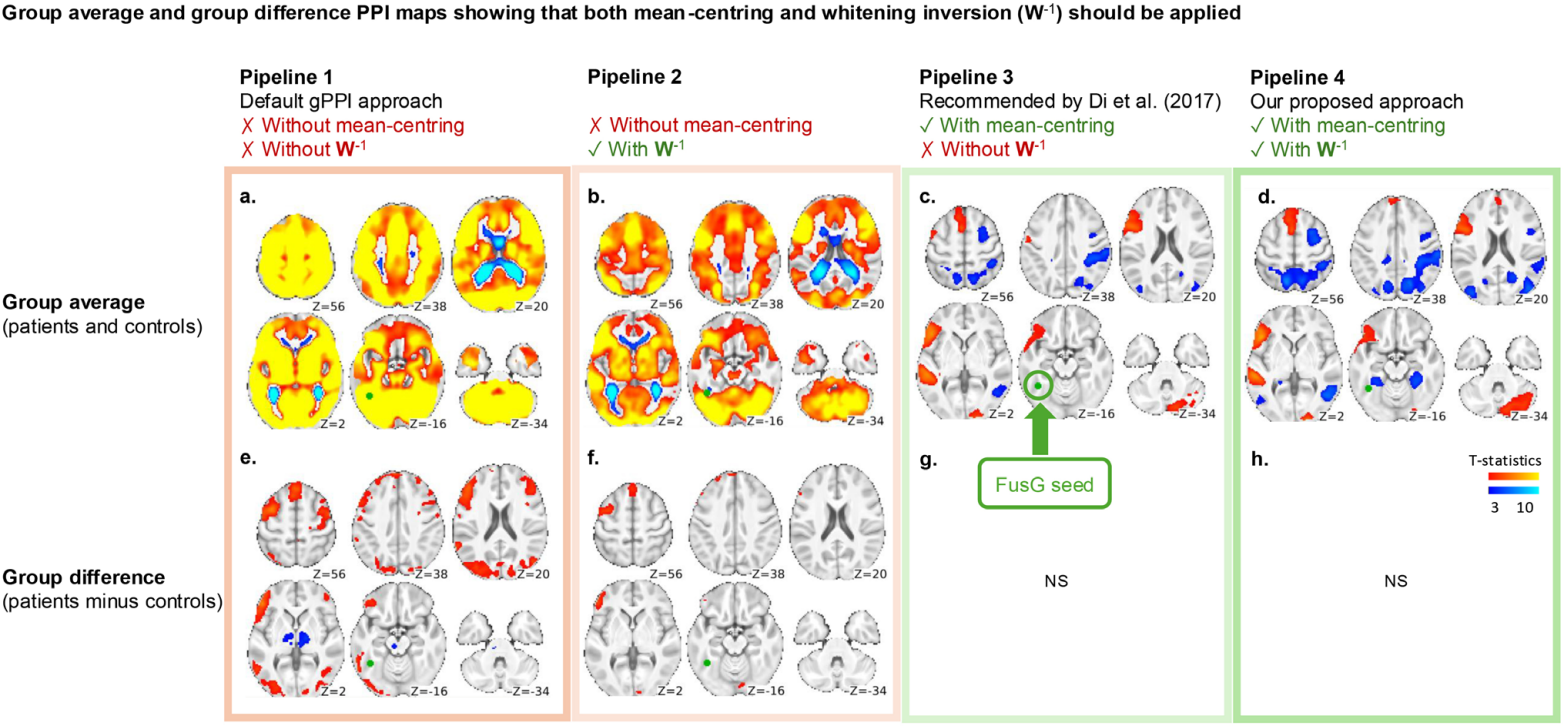
Effects of mean-centering and whitening inversion on group PPI results. Different pipelines are shown as columns. Group average and group differences in interaction effects are shown in the top and bottom rows, respectively. FusG seed location is shown in green. Left hemisphere on the left side. FWEc p < 0.05, two-sided. NS: not significant. (a) Without mean-centering and without whitening inversion (Pipeline 1), leads to unreasonably widespread and implausible interaction effects, including artefactual interaction effects at the seed location. (b) Whitening inversion (Pipeline 2) removes interaction effects at the seed location, but implausible widespread effects are still present. (c) Mean-centering (Pipeline 3) eliminates widespread interaction effects, leaving positive effects in left language areas and negative effects in right hemisphere attentional areas. (d) Combining mean-centering with whitening inversion (Pipeline 4) improves detection of interaction effects relative to mean-centering alone. Bottom row: with correct mean-centering (g, h) there are no significant differences observed between our particular groups. If only inspecting between group maps (e, f), model misspecification would not necessarily be as apparent as seen in (a, b). Unthresholded results are provided in Supplementary Figure 2. Difference maps relative to Pipeline 4 are provided in Supplementary Figure 4.

**Fig. 3.**
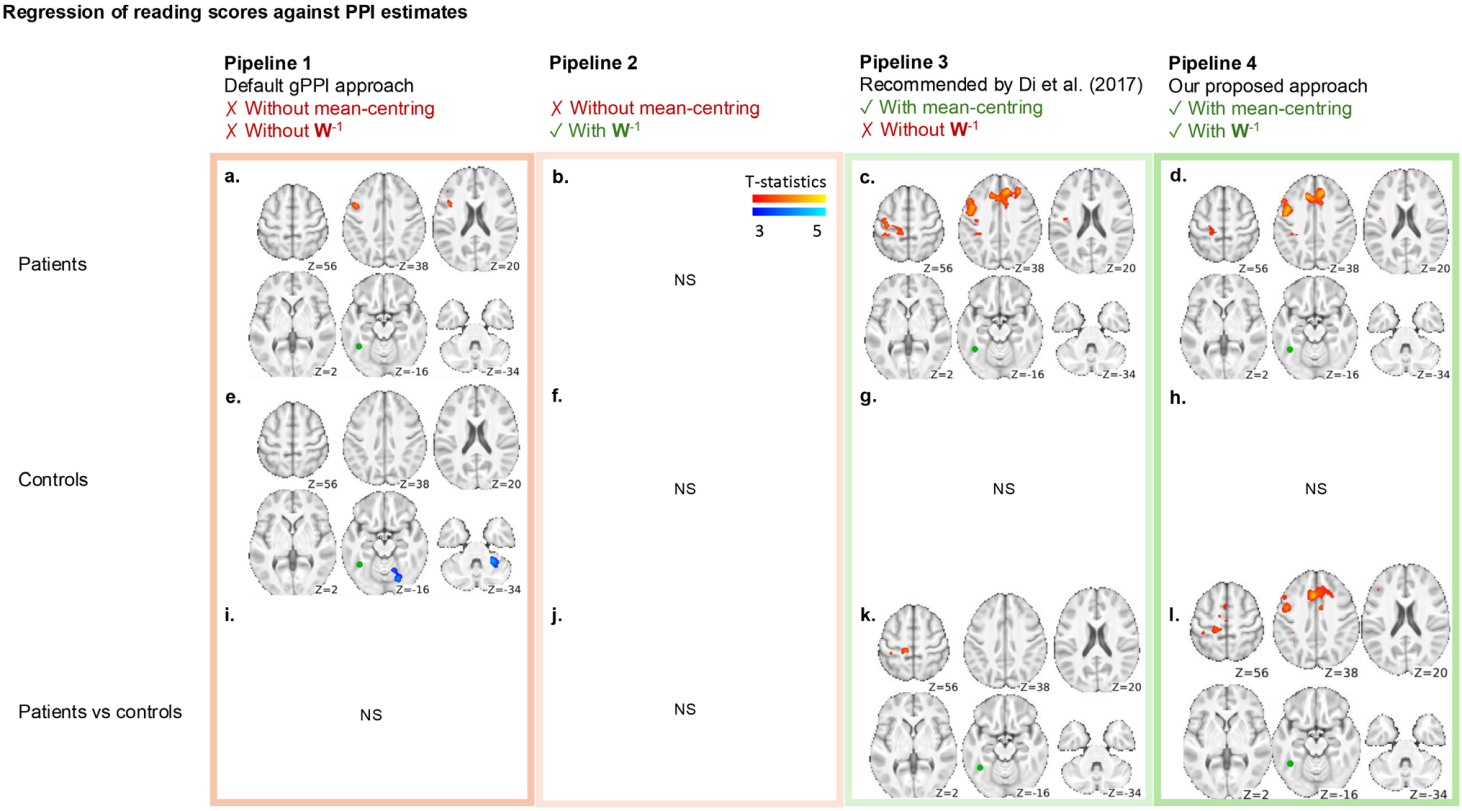
Regression of reading scores against PPI estimates. Different pipelines are shown as columns. The reference approach is to apply both mean-centering and whitening inversion (Pipeline 4). Results from Pipeline 3 (mean-centering only; c, g, k) were mostly consistent with Pipeline 4 (d, h, l). Without mean-centering (Pipelines 1 and 2; a, e, i, b, f, j), results were largely inconsistent compared to Pipeline 4. However, since neither the widespread effects nor effects at the seed location were present (see Fig. 2 and section 3.2), it may not be obvious that these results were incorrect unless a proper analysis incorporating mean-centering is performed. FusG seed location is shown in green. Left hemisphere on the left side. FWEc p < 0.05, two-sided. Unthresholded results are provided in Supplementary Figure 3. Difference maps relative to Pipeline 4 are provided in Supplementary Figure 5.

We next performed PPI analyses (Friston et al., 1997) seeding from left fusiform gyrus (FusG) to test the hypothesis that across all participants, there would be upregulation of information flow between FusG and major language nodes during pseudoword rhyming relative to pattern matching. We defined the seed region using a sphere of 6 mm radius, centered on the peak activation coordinate in left FusG from the one-sample *t*-test modeling the task activation (green spheres in Fig. 2 and Fig. 3). We then extracted the first eigenvariate of the time series across all voxels within the spherical ROI for each participant using SPM’s time series extraction function. This included applying a high-pass filter of 128 s, confound-correction, and prewhitening. All analyses were performed using SPM12 (revision 7771) and the gPPI toolbox (v13.1) with modified code (details below).

#### 2.5.1 Inversion of the whitening step and mean-centering

Both whitening inversion and mean-centering are not available within the current release version of the gPPI package (v13.1) at the time of writing. We, therefore, implemented them by modifying the relevant lines of code. For whitening inversion, we modified the *timeseries_extract* function called at line 463 of the *PPPI.m* function, changing line 397 from *xY.u = Y* to xY.u = inv(SPM.xX.W)*Y. For mean-centering, we modified line 705 in *PPPI.m* function, changing PSYxn(:,j) = PSY(:,j).*xn to PSYxn(:,j) = (PSY(:,j)-mean(PSY(:,j))).*xn. Some users perform PPI analysis directly using SPM functions instead of the gPPI toolbox. In this case, one can modify line 258 of the *spm_regions.m* function by changing *xY.u* = *Y* to *xY.u* = *inv(SPM.xX.W)*.*Y. In SPM12 (version r7771 or later), the task regressor is mean-centered by default, so when using one of these more recent versions of SPM users do not need to manually edit the code to apply mean-centering. In addition, one only needs to modify the code to mean-center the task regressor in the interaction term, as the seed regressor is mean-centered by default. We note that technically high-pass filtering is also applied twice to the seed time series in a PPI model. SPM implements filtering similar to a confound regression. Therefore, applying the filter multiple times is equivalent to regressing out the same components repeatedly, which does not affect the regression results. This time series extraction step is also entirely independent of the PPI model fitting; hence, the filtering applied during extraction does not affect the degrees of freedom of the PPI model.

We repeated the same PPI analysis both with and without whitening inversion, and with and without mean-centering. This resulted in four analysis pipelines:

**Pipeline 1**: Without whitening inversion and without mean-centering. This is the default pipeline used in the gPPI toolbox, and in PPI analysis implemented in SPM5 r3271 – SPM12 r6556.

**Pipeline 2**: With whitening inversion and without mean-centering.

**Pipeline 3**: Without whitening inversion and with mean-centering. This is the pipeline used in our previously published study (He et al., 2025) and is the approach recommended by Di et al. (2017). It is also the approach that would likely result from manual construction of a PPI analysis (i.e., without the gPPI toolbox) in SPM12 r7771 or later, as the regressors are mean-centered by default.

**Pipeline 4**: With whitening inversion and with mean-centering. This is our recommended pipeline.

#### 2.5.2 Second-level analysis

For each pipeline, one-sample *t*-tests were performed on group-averaged interaction effects in the seizure group (n = 94), controls (n = 107), and seizure and controls combined (n = 201). We also ran a two-sample *t*-test to compare PPI maps of the seizure group and controls. Finally, we ran a whole-brain mixed-effects regression model designed to investigate whether PPI parameter estimates covary with reading performance, and whether these relationships differ between the seizure group (n = 89, 2 seizure participants had missing reading scores and 3 had invalid scores) and controls (n = 94, 13 controls had invalid reading scores). Age, sex, and a constant term were included in the model as regressors of no interest. We investigated both positive and negative effects; hence, a two-tailed FWEc threshold of p < 0.05 was applied, with an initial cluster-forming threshold of p < 0.001 uncorrected.

### 2.6 A systematic survey on PPI literature from 2018 to 2022

We investigated whether mean-centering the task regressor during the generation of the interaction term, as recommended by Di et al. (2017), has become a new standard in the field. We searched the PPI literature for articles published between 1 January 2018 and 31 December 2022 on Clarivate Web of Science (https://www.webofscience.com/wos/alldb/basic-search) that have the terms “psychophysiological interaction” and “fMRI” in their title, abstract, or keywords. A total of 169 papers were identified. We excluded five papers: three due to their focus on methodology rather than the application of PPI on an actual dataset, and two because the authors did not apply PPI to their data. The final number of included papers is 164.

**Fig. 4.**
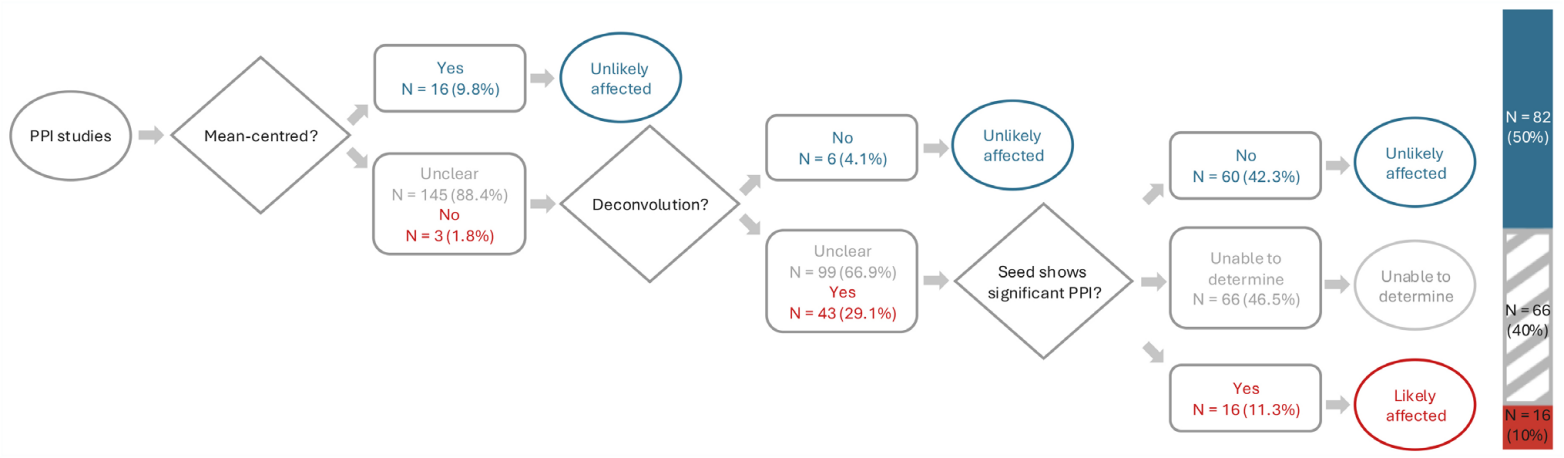
Screening flowchart. We first screened the methods section of each paper to determine whether the authors mean-centered the task regressor when calculating the interaction term. For those that did not mean center or had unclear methods, we checked whether they applied deconvolution in their PPI analysis. For those that applied deconvolution or had unclear descriptions, we screened their results for any suspicious PPI findings. Specifically, we checked whether the reported interaction effects overlapped with the seed region itself. As discussed in section 3.2, without mean-centering or whitening inversion, it is likely that the resulting interaction effects will peak at the seed location. We categorized each study as “unlikely affected”, “unable to determine”, or “likely affected” by the mean-centering issue. Percentages add up to 100% for each branch.

For each paper, we screened the methods and results to categorize them into one of three categories: “unlikely affected” by the non-mean-centering issue in PPI (see section 3 and Di et al., 2017), “likely affected” by the non-mean-centering issue, or “unable to determine”. The screening process is as follows (also see Fig. 4):

Step 1: We screened the methods to determine whether they mean-centered the task regressor when calculating the interaction term. For those that explicitly mentioned a mean-centering step, we categorized them as “unlikely affected” by the non-mean-centering issue.

Step 2: For the remaining studies, we checked whether they applied deconvolution in their PPI computations. As discussed in Di et al. (2017), mean-centering is not necessary if deconvolution is not performed. Deconvolution is not implemented in FSL (Jenkinson et al., 2012), BrainVoyager (Goebel, 2012), and the CONN toolbox (Whitfield-Gabrieli & Nieto-Castanon, 2012). Therefore, studies using only these software were categorized as “unlikely affected”.

Step 3: For studies that did not apply mean-centering (or had unclear methods) but applied deconvolution (or had unclear methods), we screened their results for any suspicious PPI findings. Specifically, we checked whether the reported interaction effects overlapped with the seed region itself. At the seed location, the principal effect should just be that of the seed regressor. While the absence of an interaction effect does not guarantee that a PPI model is correctly specified, the detection of an interaction effect at the seed location is a clear sign of model misspecification. Studies that showed significant group-averaged interaction effects in the seed region were, therefore, categorized as “likely affected”. Studies that did not show significant group-averaged interaction effects in the seed region were categorized as “unlikely affected”. Studies were categorized as “unable to determine”, if (1) they consisted of group comparisons and did not report group-averaged PPI effect maps, because some or all of the effect on the seed region might cancel out (as we show using our own data in section 3.2), or (2) they consisted of behavioral correlations and did not report group-averaged PPI effect maps, because the effect on the seed region might not be captured in the behavioral regressor (see section 3.2), or (3) they consisted of ROI-to-ROI analysis and did not report the correlation with the seed itself, or (4) they reported group-averaged PPI maps but were unclear about the location of the seed in relation to the PPI maps.

## 3 Results

### 3.1 Prewhitening adversely affects deconvolution-based estimation of the underlying neuronal signal

We first demonstrated, via simulations, that prewhitening adversely affects the deconvolution process (Fig. 1b). With whitening inversion, which is equivalent to removing prewhitening in this case (as there is no confound regression involved), the deconvolved signal closely aligns with the simulated neural signal.

### 3.2 Group-level results

#### 3.2.1 Whitening inversion removes spurious interaction effects at the seed location even without mean-centering

Without mean-centering (Pipelines 1 and 2), we observed widespread group-averaged positive interaction effects extending well beyond areas that might reasonably be hypothesized to be involved (Fig. 2a, b). The spurious effects are lessened with whitening inversion (Pipeline 2, Fig. 2b), which also eliminates artefactual interaction effects at the seed location.

#### 3.2.2 Mean-centering eliminated widespread interaction effects, while whitening inversion enhanced the clusters

With mean-centering (Pipelines 3 and 4), the widespread spurious interaction effects seen in Pipelines 1 and 2 were eliminated. Significant interactions during rhyming were limited to language regions, and during pattern matching to right parietal and posterior temporal regions (Fig. 2c, d). In addition, there is no between-group difference in interaction effects (Fig. 2g, h). Without mean-centering (Pipelines 1 and 2), the strong and widespread interaction effects present in each group individually (see Supplementary Fig. 1 for group specific results) largely cancel out, as do the artefactual effects at the seed location; some spurious group differences, nevertheless, remain (Fig. 2e, f). In other words, if only examining between group effects, the consequences of model misspecification may not be obvious.

#### 3.2.3 Impact of PPI model misspecification on behavioral regression

Taking Pipeline 4 as the reference processing approach, we found a positive relationship between reading scores and the PPI parameter estimates in the superior medial frontal region and the left middle frontal gyrus, in the seizure patient group (Fig. 3d). In contrast, no such relationship was observed in controls (Fig. 3h), and the difference was significant between patients and controls (Fig. 3l). If only mean-centering was applied without whitening inversion (Pipeline 3), the results appeared broadly comparable (Fig. 3c, g), though the group differences diminish (Fig. 3k). Without mean-centering (Pipelines 1 and 2), results are either missed (Fig. 3b), diminished (Fig. 3a), or spurious (Fig. 3e).

### 3.3 PPI mean-centering error remains an issue in 10% or more of published studies

We have shown that in deconvolution-based PPI models mean-centering reduces the likelihood of spurious interaction effects and whitening inversion increases power to detect true interaction effects. Given Di alerted the field to the mean-centering issue in 2017, and the risk of false positive findings from failing to mean-center, we surveyed PPI studies published between 2018 and 2022 to investigate contemporary adoption of the mean centering approach. Among the 164 studies identified, we categorized 16 (10%) as “likely affected”, 66 (40%) as “unable to determine”, and 82 (50%) as “unlikely affected” by this non-mean-centering issue in PPI. Thus, 10% or more of recently published studies using PPI methods are likely based upon misspecified models (Fig. 4).

## 4 Discussion

Through both simulation and the analysis of real data, we have demonstrated two issues in the implementation of PPI analysis in SPM and the gPPI toolbox, one previously reported—mean-centering, and the other novel—prewhitening. The practical consequences of these two issues on group-averaged interaction effects are highlighted in Figure 2a-d. Comparing Figure 2a or Figure 2b with Figure 2c demonstrates that mean centering is essential to avoid false-positive interaction effects regardless of the prewhitening issue. We expect this, because mean-centering ensures there is no main effect seed component in the PPI term (Di et al., 2017). However, comparing Figure 2a with Figure 2b reveals prewhitening can worsen the false-positive interaction effects that result from a lack of mean-centering, including emergence of false-positive PPI at the seed location. Finally, comparing Figure 2c with Figure 2d demonstrates the optimum model (Pipeline 4; where the seed regressor is better represented in the model) confers additional power to detect true interaction effects.

We further showed that group comparisons can conceal some of these effects, and that model misspecification has deleterious consequences for detecting behavioral correlations. In a systematic survey of the recent literature, we discovered that PPI model misspecification remains a non-negligible problem in contemporary work.

### 4.1 Presence of a significant interaction effect at the seed location as a ‘red flag’

The presence of a significant interaction effect at the seed location itself is a telltale sign of model misspecification. We have shown that including a whitening inversion step ensures that the main effect seed regressor in the PPI model is well-matched to the signal at the seed location itself, preventing the emergence of spurious interaction effects at the seed location (Fig. 2b). We note that an alternative approach is to skip prewhitening in time series extraction, as the regression coefficients in confound regression should remain asymptotically unbiased despite the presence of autocorrelation (Gauss-Markov Theorem; see also Arbabshirani et al., 2014; Friston et al., 2007; Olszowy et al., 2019). However, skipping this step would require significant code changes, as SPM’s confound regression relies on prewhitened first level beta maps. Those beta coefficients are estimated with prewhitening. It is not possible to obtain beta coefficients without prewhitening unless the maximum likelihood estimation is redone, which would require writing a new function to refit the GLM. Alternatively, one may achieve this by additionally performing a first-level analysis without specifying an autocorrelation model and then extract time series from its output.

### 4.2 Consequences of not mean-centering

If mean-centering is not performed, the interaction term carries a seed component that does not match perfectly with the main effect seed regressor due to the deconvolution algorithm, whereas mean-centering avoids this component (Di et al., 2017). To understand the potential consequences of mean-centering or not, we first compared the patterns of results that follow from these two approaches when applied to our own data. Using a deconvolution approach with proper mean-centering, we observed task-dependent connectivity from left FusG to language and visuospatial attentional regions, in both seizure and control participants (Fig. 2; details in He et al., 2025). Without mean-centering, none of the PPI maps are valid due to the extra unwanted variance in the interaction term that highly correlates with the main effect of the seed (Figs. 2 and 3). The consequences of the misspecification are widespread and obvious when we look at group-averaged interaction effects, but less obvious in the group comparison map and the second-level regression maps (first two columns Figs. 2 and 3)—it becomes evident that these maps cannot be trusted when compared to the correctly mean-centered results (last two columns Figs. 2 and 3).

### 4.3 Survey of contemporary practice

In the systematic survey of recently published PPI literature, we noticed many studies fall into the “unable to determine” category due to insufficient details provided in the paper. For example, many papers neither indicated whether mean-centering was applied nor specified how the task regressor was coded. Certain versions of SPM, and the gPPI toolbox, do not apply mean-centering in PPI models, and it is impossible to extract this information if the software version number is not included in the paper. In a misspecified PPI model, there are likely spurious interaction effects at the seed location itself, and potentially also in brain areas correlated with the seed, as discussed in section 3.2.1, Di et al. (2017), and Masharipov et al. (2024). Therefore, for studies with unclear methods regarding whether mean-centering and/or deconvolution were applied, we needed to screen their results to check whether the reported interaction effects overlap with the seed. During this process, often we found it unclear where the seed is located on the PPI maps. We also note that comparing groups or performing second-level regressions without considering the PPI results at the single group level can make it challenging to detect abnormalities. In addition, some studies adopted an ROI-to-ROI approach to increase power, as the effect size of the interaction term may be much smaller than that of the main effects (O’Reilly et al., 2012). In this case, if the authors do not check the interaction with the seed region itself, the results can be difficult to assess.

As a side note, we noticed that the modal sample size used in the 164 PPI papers reviewed in this study was 23 (IQR = 19.75). Based on our own analysis, the interaction term has an effect size of approximately 0.4 (calculated by dividing the mean beta estimates across each cluster by the standard deviation). To achieve power of 0.8, at least 52 participants are required (Faul et al., 2007), which is consistent with a simulation-based study by Masharipov et al. (2024). Therefore, most of the included studies may be underpowered. Without proper mean-centering, the significant results reported in PPI studies could be borrowing from the main effects, as demonstrated in our empirical fMRI analysis (see Fig. 2a).

### 4.4 Recommendations for implementing and reporting PPI analysis

We propose several recommendations for conducting PPI analyses. Firstly, it is important for users to understand how an analysis has been implemented in the software being used. In the context of PPI, one should be aware of how a time series is extracted, as well as whether mean-centering has been applied by default and whether a deconvolution step is involved. After implementing a PPI analysis, a quick sanity check is to screen the interaction effect in the seed region. A misspecified PPI model is likely to show a significant interaction effect in the seed, as discussed in section 3.2.1 and Di et al. (2017). For group comparisons, one should check the PPI maps at a single group level. For ROI-to-ROI analysis, one should check the interaction effects with the seed ROI itself. It is also important to note that non-mean-centered PPI results may consist of a combination of correct and spurious interaction effects. It would, therefore, be challenging to disentangle these effects without conducting a proper PPI analysis with mean-centering.

When reporting a PPI analysis, authors should detail whether mean-centering and deconvolution were applied, as well as the software version used to implement the analysis (Nichols et al., 2016). In the results section, authors should report their PPI maps with reference to location of the seed (e.g., by showing slices that intersect with the seed, and with the seed indicated). In studies with multiple group comparisons or second-level regressions, it may be useful to also include single-group PPI maps even if only as supplementary information. At the very least, authors should review their own single-group maps as a data quality control step, to check for spurious interaction effects that intersect with the seed location—a sure sign of model misspecification. In studies with ROI-to-ROI analysis, authors should also report the interaction effect with the seed ROI. We summarized all the suggested steps in a checklist (Table 1).

**Table 1.** Checklist for implementation and reporting of PPI analyses.

Stage	Item	Desired outcome	Consequences
Before running the analysis	1. Does the software apply prewhitening during time series extraction?	No	If *yes*, turn off this option (if possible) or modify the code to either (i) prevent prewhitening or (ii) implement whitening inversion.
	2. Does the software mean-center the regressors when generating the interaction term?	Yes	If *no*, modify the code to mean-center the regressors when generating the interaction term.
	3. Does the software apply deconvolution?	Yes/No	If *yes*, check to make sure mean-centering is applied.
	4. Are you using the gPPI toolbox (v13.1 or earlier versions)?	Yes/No	If *yes*, modify the code to implement mean-centering and whitening inversion as described in section 2.5.1.
After running the analysis	5. Check individual PPI maps and group-averaged PPI maps. Is a significant interaction (PPI) effect observed at the seed location?	No	If *yes*, check if mean-centering and whitening inversion have been applied. Interaction effects should not be seen at the seed location. If they are, this suggests model misspecification.
Reporting: in Methods	6. Are details on prewhitening, mean-centering, and deconvolution reported?	Yes	For reproducibility.
Reporting: in Results	7. Are PPI maps reported with reference to location of the seed, by showing slices that intersect with the seed, and with the seed indicated?	Yes	For reviewing of PPI results.
	8. If group-averaged interaction effects are not included in main results, are they at least included as supplementary information?	Yes	For reviewing of PPI results.

### 4.5 Conclusion and future work

In conclusion, we identified two issues associated with conventional PPI approaches that use deconvolution: one with prewhitening and one with mean-centering. We demonstrated that these errors are non-negligible through both simulations and applications to empirical data. Notably, such model misspecification can be easily overlooked if one examines only between-group interaction effects. Additionally, a systematic survey of recent literature revealed that model misspecification and insufficient methods reporting remain significant issues in the field. We have made contact with the SPM team, and they are working on a new function to preclude prewhitening in time series extraction (see GitHub issue: https://github.com/spm/spm/issues/68). The TMFC toolbox (Masharipov et al., 2024) has also implemented a whitening inversion option via a graphical user interface in response to the preprint of this paper. It is also worth noting that Dynamic Causal Modelling (DCM; Friston et al., 2003) also has an implicit deconvolution step, and future research could investigate whether prewhitening also affects DCM. Our findings show that PPI is a valuable technique for interrogating brain networks in vivo, but highlight the importance of proper model specification to ensure appropriate inference.

## Supplementary Material

Supplementary Material

## Data Availability

Any requests for access to the data used in this project should be directed to the Australian Epilepsy Project (a formal data access request can be lodged at https://www.epilepsyproject.org.au/research/access-to-aep-data). The analyses were conducted using publicly available software, and no custom code was developed other than the two modifications described in the manuscript.
