# Supplementary material for: Common pitfalls during model specification in psychophysiological interaction analysis
Source: Imaging Neurosci (Camb). 2025 Nov 10;3:IMAG.a.989. doi: 10.1162/IMAG.a.989 (PMC12603652; doi:10.1162/IMAG.a.989)
Supplement: Supplementary Material [file IMAG.a.989_supp.pdf]

Supplementary Information

Common pitfalls during model specification in psychophysiological interaction analysis

Vicky He*[1,2], Bahman Tahayori[1,2], David N. Vaughan[1,2,3], Heath R. Pardoe[1,2], Graeme D. Jackson[1,2,3], Chris Tailby[†,*1,2,4], and David F. Abbott[†,*1,2,5]

[1]The Florey Institute of Neuroscience and Mental Health, Heidelberg, Victoria, Australia

[2]Florey Department of Neuroscience and Mental Health, The University of Melbourne, Parkville, Victoria, Australia

[3]Department of Neurology, Austin Health, Heidelberg, Victoria, Australia

[4]Department of Clinical Neuropsychology, Austin Health, Heidelberg, Victoria, Australia

[5]Department of Medicine - Austin Health, The University of Melbourne, Heidelberg, Victoria, Australia

[†]Joint senior authors

*Email for correspondence: he.v@unimelb.edu.au; david.abbott@florey.edu.au; chris.tailby@florey.edu.au

List of Contents

Supplementary Figures 1-5

Supplementary Table 1

Supplementary Figure 1

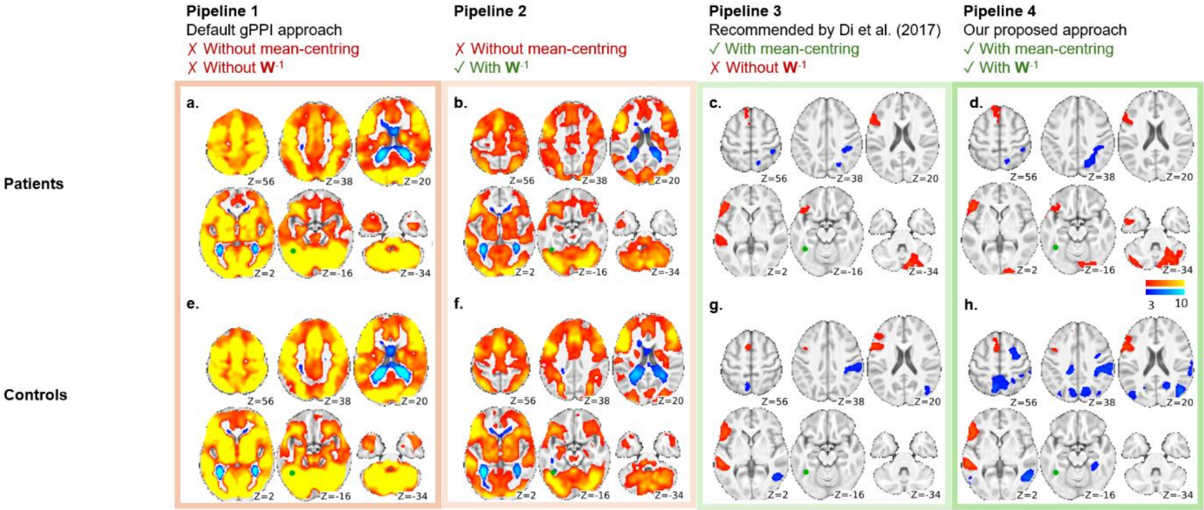

Fig. 1. Effects of mean-centring and whitening inversion on group specific PPI results. Different pipelines are shown as columns. Patient group and control group interaction effects are shown in the top and bottom rows, respectively. FusG seed location is shown in green. Left hemisphere on the left side. FWEc $p < 0.05$, two-sided. Direct comparisons of patients versus controls are shown in the manuscript Figure 2; no significant between group differences are observed when using Pipelines 3 or 4.

Supplementary Figure 2

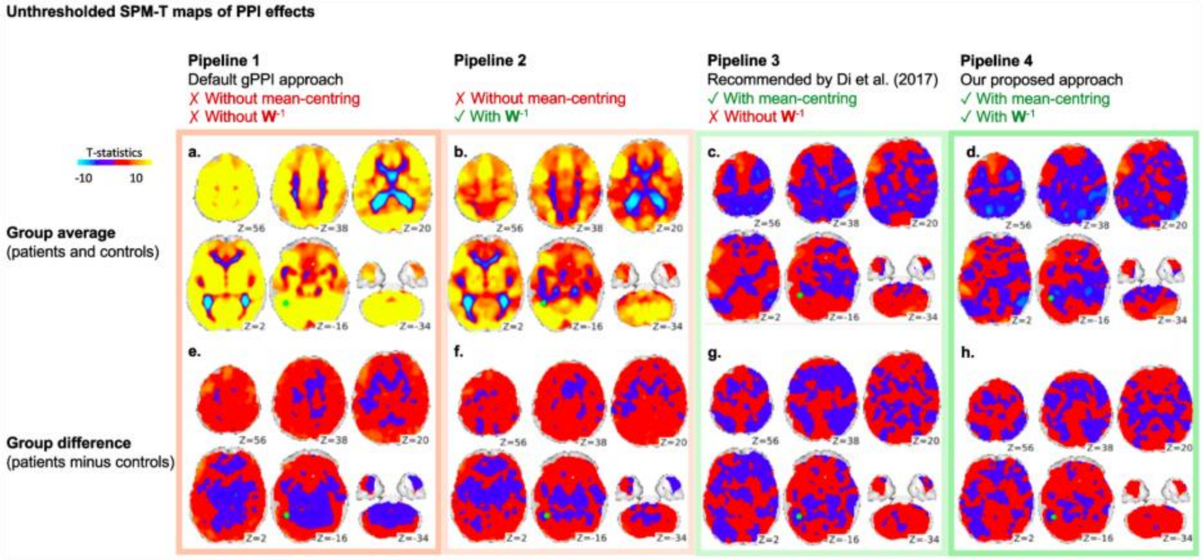

Fig. 2. Unthresholded SPM-T maps on group PPI results. Different pipelines are shown as columns. Group average and group differences in interaction effects are shown in the top and bottom rows, respectively. FusG seed location is shown in green. Left hemisphere on the left side.

Supplementary Figure 3

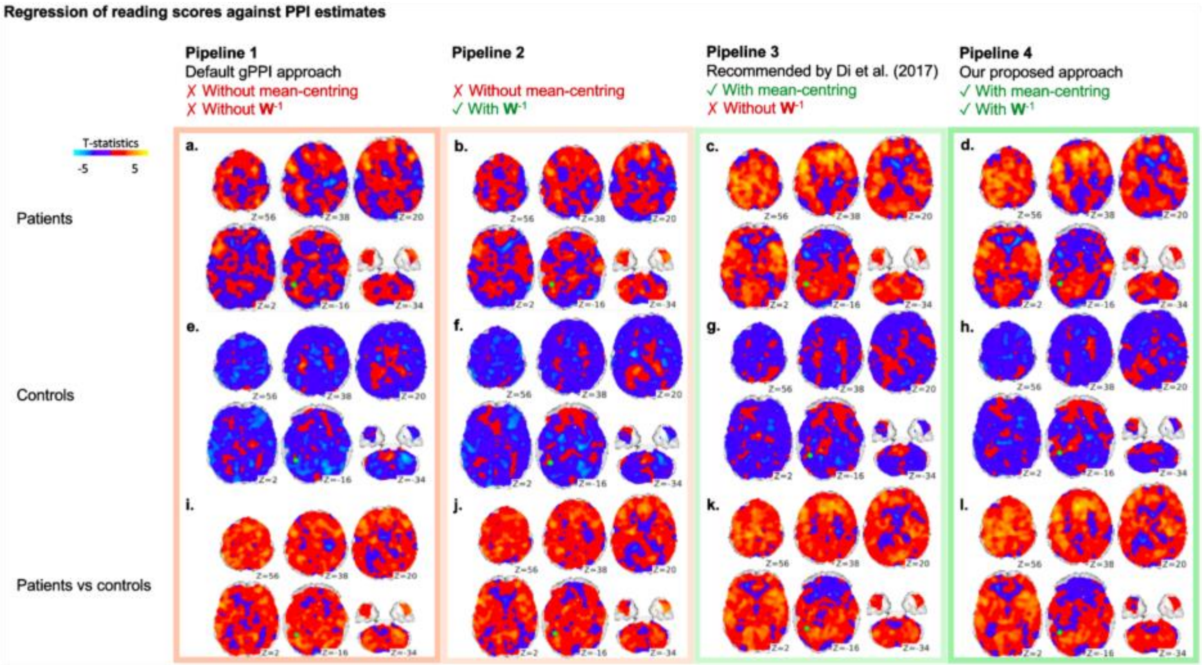

Fig. 3. Unthresholded SPM-T maps on regression coefficients of reading scores against PPI estimates. Different pipelines are shown as columns. FusG seed location is shown in green. Left hemisphere on the left side.

Supplementary Figure 4

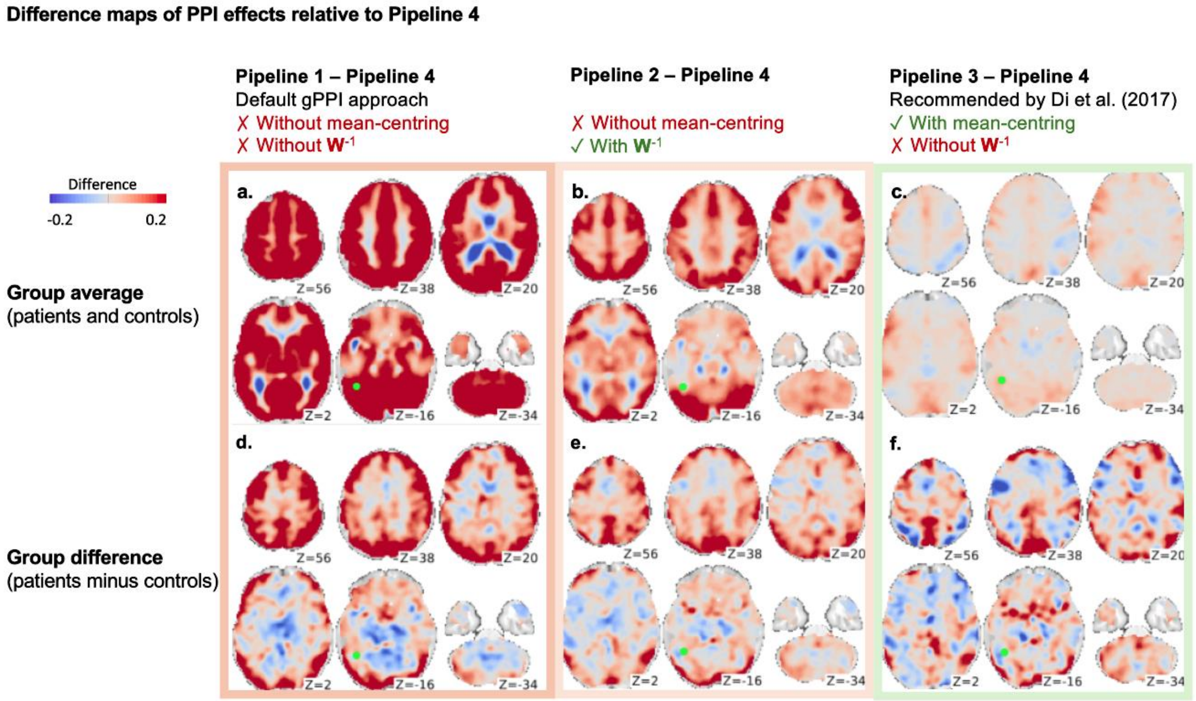

Fig. 4. Difference maps on group PPI results obtained by subtracting the beta maps of pipeline 4 from those of pipelines 1-3. Different pipelines are shown as columns. Group average and group differences in interaction effects are shown in the top and bottom rows, respectively. FusG seed location is shown in green. Left hemisphere on the left side.

Supplementary Figure 5

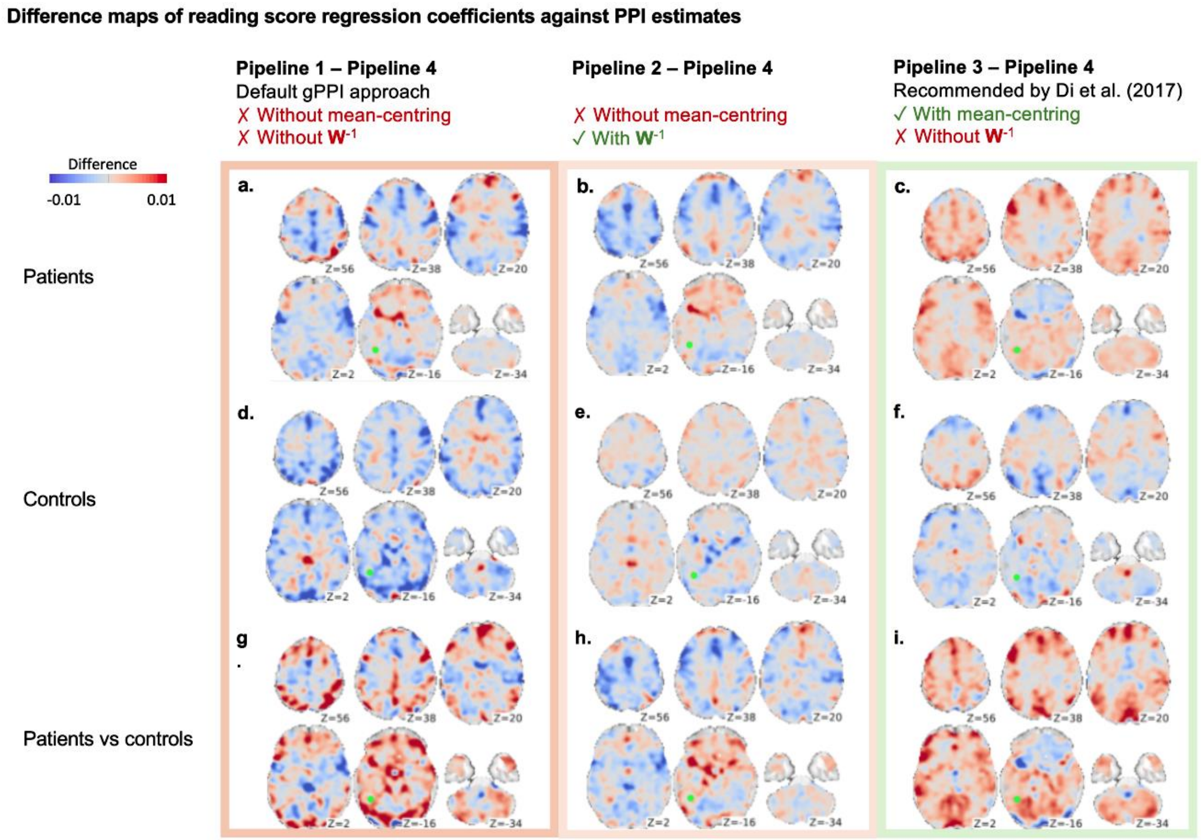

Fig. 5. Difference maps on regression coefficients of reading scores against PPI estimates obtained by subtracting the beta maps of pipeline 4 from those of pipelines 1-3. Different pipelines are shown as columns. FusG seed location is shown in green. Left hemisphere on the left side.

Supplementary Table 1. AEP investigator list with Contributor Roles Taxonomy (CRediT) author statement relevant for this manuscript.

Australian Epilepsy Project Investigators			
**Name & ORCID**	**Primary Location**	**Role**	**CRediT**
**Graeme D. Jackson, MD 0000-0002-7917-5326**	The Florey Institute of Neuroscience and Mental Health	Chief Investigator	Conceptualisation, Methodology, Investigation, Resources, Writing - Review & Editing, Supervision, Project Administration, Funding Acquisition
**David F. Abbott, PhD 0000-0002-7259-8238**	The Florey Institute of Neuroscience and Mental Health	Informatics Lead	Conceptualisation, Methodology, Software, Investigation, Resources, Data Curation, Writing - Original Draft, Writing - Review & Editing, Visualisation, Supervision, Project Administration, Funding Acquisition
**Zanfina Ademi, PhD**	Monash University	Health Economics Lead	Conceptualisation, Funding acquisition

**0000-0002-0625-3522**			
**Subhaga Amarasekara**	The Florey Institute of Neuroscience and Mental Health	Product Lead	Resources, Project Administration
**Amanda Anderson**	The Florey Institute of Neuroscience and Mental Health	Lived Experience Ambassador and Participant Lead	Investigation, Resources, Funding acquisition
**Rachel Hughes**	The Florey Institute of Neuroscience and Mental Health	Clinical Research Coordinator	Investigation, Resources
**Donna Hutchison**	The Florey Institute of Neuroscience and Mental Health	Executive Lead	Project administration
**Patrick Kwan, MD 0000-0001-7310-276X**	Monash University	Outcomes Lead	Conceptualisation, Resources, Funding acquisition
**Paul Lightfoot**	The Florey Institute of Neuroscience and Mental Health	Operations Lead	Investigation, Project administration
**Saul Mullen, MD, PhD 0000-0003-1224-4101**	The University of Melbourne	Protocol Development Lead (2019-2021)	Conceptualisation, Methodology, Funding acquisition
**Karen L. Oliver, PhD 0000-0001-5188-6153**	The University of Melbourne	Genetics Lead	Conceptualisation, Funding acquisition

**Heath R. Pardoe, PhD 0000-0002-0123-2167**	The Florey Institute of Neuroscience and Mental Health	Science Operations Lead	Investigation, Resources, Writing - Review & Editing, Project Administration
**Mangor Pedersen, PhD 0000-0002-9199-1916**	Auckland University of Technology	Artificial Intelligence Lead	Conceptualisation, Methodology, Funding acquisition
**Chris Tailby, PhD 0000-0002-1320-5924**	The Florey Institute of Neuroscience and Mental Health	Neuropsychology Lead	Conceptualisation, Methodology, Investigation, Resources, Data Curation, Writing - Original Draft, Writing - Review & Editing, Visualisation, Supervision, Project Administration, Funding Acquisition
**David N. Vaughan, MD, PhD 0000-0002-6225-7739**	The Florey Institute of Neuroscience and Mental Health	Imaging Lead	Conceptualisation, Methodology, Investigation, Resources, Writing - Review & Editing, Project

			Administration, Funding Acquisition
**Anton De Weger 0009-0006-7478-361X**	The Florey Institute of Neuroscience and Mental Health	Digital and Technology Lead	Software, Resources, Data Curation
